# Effect of Co-culturing both placenta-derived mesenchymal stem cells and their condition medium in the cancer cell (HepG2) migration, damage through apoptosis and cell cycle arrest

**DOI:** 10.1016/j.sjbs.2022.103519

**Published:** 2022-12-01

**Authors:** F.A. Dain Md Opo, Mohammed Moulay, Ghadeer I. Alrefaei, Nouf H. Alsubhi, Saleh Alkarim, Mohammed M. Rahman

**Affiliations:** a Department of Biological Science, Faculty of Sciences, King Abdulaziz University, Jeddah 21589, Saudi Arabia; b Embryonic Stem Cell Research Unit, King Fahd Medical Research Center, King Abdulaziz University, Jeddah 21589, Saudi Arabia; c Department of Biology, College of Science, University of Jeddah, Jeddah 21589, Saudi Arabia; d Biological Sciences Department, College of Science & Arts, King Abdulaziz University, Rabigh 21911, Saudi Arabia; e Embryonic and Cancer Stem Cell Research Group, King Fahd Medical Research Center, King Abdulaziz University, Jeddah 21589, Saudi Arabia; f Department of Chemistry, King Abdulaziz University, Jeddah 21589, Saudi Arabia

**Keywords:** HepG2, hPMSCs, Apoptosis, Morphology, Differentiation, Conditioned medium, AFP, Alfa Feto Protein, hP-MSCs, human Placenta derived Mesenchymal Stem Cells, HCC, Hepatocellular Carcinoma, IL, Interleukin, PVDF, Polyvinylidene Fluoride, TBST, Tris-Buffered Saline with 0.1% Tween 20 detergent., 70P, 70% placenta, 50P, 50% placenta, 30P, 30% placenta, 70H, 70% HepG2, 50H, 50% HepG2, 30H, 30% HepG2

## Abstract

Human placental-derived mesenchymal stem cells (hPMSCs) are a promising candidate to inhibit the proliferation of hepatocellular carcinoma (HCC) cell lines such as HepG2. The effects of hPMSCs and their conditioned media on HepG2 are, however, still a mystery. As a result, the goal of this study was to look into the effects of hPMSCs and their conditioned media on HepG2 and figure out what was going on. Fluorescence-activated cell sorting and the MTT test were used to determine the percentage of cells that died (early apoptosis, late apoptosis). The DIO and DID colors were used to detect cell fusion and cell death in both cells. HepG2 cells were co-treated with hPMSCs or hPMSCs-conditioned medium (hPMSCs-CM) to reduce growth and promote apoptosis. Morphological changes were also seen in the 30 percent, 50 percent, and 60 percent cases. The secretion of cytokine was determined by the ELISA. Flow cytometry, caspase 9 immunofluorescence, qPCR (detection of Bax, Bcl-2, and β-catenin genes), western blot, and immunophenotyping revealed that treatment with hPMSCs or hPMSCs-CM caused HepG2 cell death through apoptosis (detection of caspase 9, caspase 3 protein). HepG2 cell cycle arrest could be induced by hPMSCs and hPMSCs-CM. Following treatment with hPMSCs or hPMSCs-CM, HepG2 cell development was stopped in the G0/G1 phase. These treatments also inhibited HepG2 cells from migrating, with the greatest effect when the highest ratio/concentration of hPMSCs (70%) and hPMSCs-CM were used (90%). Our findings indicated that hPMSCs and hPMSCs-CM could be promising treatment options for liver cancer. To elucidate the proper effect, more research on liver cancer-induced rat/mice is needed.

## 1 Introduction

Hepatocellular carcinoma (HCC) is fatal cancer that can spread easily from the liver to other organs. HCC is the leading primary liver cancer that accounts for 90 % of all cases (Sia et al., 2017). It has become the sixth most frequent malignant disease globally and the second largest cause of cancer-related death (Waller et al., 2015, World Health Statistics, n.d.*: Monitoring Health for the SDGs - World | ReliefWeb*, n.d.). Viral infection, aflatoxicosis, drinking alcohol, and increasing fat in the liver are among the most common risk factors of HCC (Papatheodoridis et al., 2015). Once HCC is developed, it is not easy to be treated. However, some treatment approaches were used to extend the patient life using surgery, chemotherapy, liver transplantation, and radiotherapy. Despite progress in early diagnosis and treatment approaches for HCC in the last decades, the maximum survival time in advanced cases did not exceed 5-year due to increased metastases and high recurrence rate after treatment (W. Wang et al., 2010). Sorafenib is the first orally active multi-kinase inhibitor and the only licensed molecular targeted drug against HCC. However, its usage has been limited due to current safety concerns. Cutaneous toxicity, gastrointestinal (GIT) troubles, loss of appetite, dizziness, vomiting, weight loss, increased blood pressure, hemorrhage, and heart attack are the most common adverse effects of sorafenib (Abdel-Rahman and Lamarca, 2017, Han et al., 2016). Hepatic artery infusion-based chemotherapy was recently used in combination with cisplatin, 5-fluorouracil, and doxorubicin to cure the advanced stage of liver cancer. However, this approach increased the chance of secondary infection. Therefore, novel therapeutic strategies for advanced or metastatic HCC are urgently required. Stem cell-based therapy would be a new pathway to treat different types of cancer, including HCC, as they can migrate into the infection site and inhibit cancer cell proliferation (CJ et al., 2016). However, the effectiveness of these cells within the hostile tissue of the tumor remains a major challenge. Tumor microenvironment rich in free radical and oxidative stress markers but lacks oxygen could inhibit stem cell proliferation and differentiation, thereby reducing their treatment capability (Ikeda et al., 2008, Motawi et al., 2014; Z. Zhang & Wang, 2013). Mesenchymal stem cells (MSCs) are multipotent cells that can self-renew, differentiate into several lineages, migrate, and interact with cancer cells. MSCs can communicate with tumor cells directly or through secreted substances (chemokines, growth factors, cytokines) (Rhodes et al., 2010). However, there are contra-dictions regarding the effects of MSCs on tumor cells. Some reports found an inhibitory effect (tumor suppression), while others denoted a stimulatory effect (tumor progression and metastasis) (Folcuti et al., 2015). MSCs can repress the proliferation and metastasis of many cancer cells, including breast, lung, colon, and liver (Alzahrani et al., 2018, Khakoo et al., 2006, Mohamed et al., 2019, Rhodes et al., 2010). MSCs arrest cancer cells in the G0/G1 phase of the cell cycle, inhibit β‑catenin, c‑Myc, and Wnt signaling, trigger apoptosis, inhibit angiogenesis, and activate macrophages (Otsu et al., 2009, Yuan et al., 2018). Aside from these studies, there are also disagreements on whether hMSCs are involved in tumor growth and progression (Hou et al., 2014). The uses of human placenta derived mesenchymal stem cells (hPMSCs) are getting popular in regenerative medicine as they can produce paracrine effects and can be used as a cellular vehicle due to their migration and tropism properties (Kam et al., 2020). HPMSCs can also differentiate into chondrocytes, osteocytes and repair the damaged tissues in the body (Ciavarella et al., 2010). Using hPMSCs is better than bone marrow-derived MSCs because their isolation is easier (non-invasive) and less immune rejection and tumor transformation. MSCs -conditioned medium (MSCs-CM) is the substances (mostly cytokines, growth factors, and extracellular vesicles) secreted by MSCs into the culture medium. According to reports, MSCs-CM has anti-tumor capabilities to inhibit cancer cell proliferation (Lu et al., 2008). Human bone marrow (hBM)-derived MSCs-CM can inhibit HepG2 cell proliferation through targeting Wnt signaling pathway (Hou et al., 2014, Shah, 2012). HBMMSCs-CM can also synergistically potentiate the anti-HepG2 effect of sorafenib (Seyhoun et al., 2019a). Moreover, hBM-MSCs secreted factor Dkk1 induces breast cancer MCF-7 cells viability loss (Qiao, Xu, Zhao, Ye, et al., 2008). MSCs-CM has also been reported to suppress ovarian tumor cells (SK-OV-3) by down regulating IGFs, IL-8, and VEGF (Cho et al., 2009). The crosstalk between cancer cells and various MSCs found in the tumor microenvironment has been shown to play a critical role in the behavior and propagation of tumors. It has been reported that CM extracted from hPMSCs reduces brain tumor cell proliferation but has less cytotoxic effect than hPMSCs (Folcuti et al., 2015). However, the differential effects of hPMSCs and their conditioned media and lysates on HepG2 cancer cells are still elusive. It is also unknown whether MSCs can kill HepG2 cells by direct contact or releasing factors abundant in their CM. Therefore, this study aimed to investigate and compare the effects of hPMSCs and their conditioned media on liver cancer HepG2 cells and elucidate the underlying mechanism of action.

## 2 Materials and methods

### 2.1 HepG2 culture

Hepatocellular carcinoma HepG2 cells (ATCC, USA; Cat. no. HB-8065) were cultured in a tissue culture flask (T25 cm^2^, Greiner) containing Dulbecco’s modified Eagle’s medium (DMEM, Gibco, USA; Cat.no.11995073) supplemented with 10 % Fetal Bovine Serum (Gibco, USA; Cat.no.10099133) and were incubated at 37 °C in a CO_2_ incubator (Life Technologies, Carlsbad, California, USA) until reaching 90 % confluence. The cells were then detached using 0.25 % Trypsin-EDTA (GIBCO, USA; Cat.no. 25200056), and the live cells were transferred in DMEM and repeated in five more flasks.

### 2.2 Human placenta mesenchymal stem Cell’s (hPMSCs) isolation and culture

The experimental procedure of this study was reviewed and approved by the Biomedical Research Ethics Committee at the Faculty of Medicine, King Abdulaziz University, Jeddah, Saudi Arabia. hPMSCs were isolated from placenta of the Saudi national women after child birth. After obtaining informed consent from all women enrolled in this study, placenta tissue was collected from 3 women (25–27 years old) after delivery at King Abdulaziz University Hospital (KAUH). Potential confounders such as gestational diabetes, preeclampsia, viral and bacterial infection were all ruled out. The specimen has been processed within 4–6 h after delivery. In brief, following washing in PBS, placenta specimens were cut into small pieces, and cells were dissociated by adding 0.1 % collagenase type I (Sigma Aldrich, St. Louis) followed by incubation (37 °C/15 min). After that, DMEM with-out FBS was added to inactivate collagenase. Following centrifugation, the cells were plated in FBS free DMEM, incubated (37 °C and 5 % CO_2_) for 10 days. MSCs from passage 4 were used in this study upon reaching 70 %–80 % confluence.

### 2.3 Preparation of hPMSCs conditioned medium

When cultured hPMSCs reach a confluence of 90 %, DMEM media without FBS was collected in a 15 ml tube and centrifuged for 5 min at 1200 rpm. The clear supernatant (hPMSCs conditioned medium) was collected after 72 h of incubation and transferred to another tube, filtered using syringe filters (0.22 µm). The hPMSCs-CM was kept at −20 °C (for immediate use) or −80 °C (for long-term use).

### 2.4 Experimental design

HepG2 cells were divided into 3 groups: the 1st group was the untreated (control) HepG2, the 2nd group in which HepG2(H) was treated with hPMSCs(P) at different ratios (30 %P:70 %H, 50 %P:50 %P, and 70 %P:30 %H), and the 3rd group in which cells were treated with hPMSCs-CM at different ratios (35 % − 50 %).

### 2.5 Cell fusion assay

DIO and DID dyes (Vybrant™ Multicolor Cell-Labeling Kit, V22889) were used to assess cell fusion between HepG2 and hPMSCs as previously described (Lee et al., 2019). Briefly, HepG2 cells (1 × 10^6^) labeled with DIO and hPMSCs (1 × 10^6^) labeled with DID were incubated for 30 min at 37 °C and then were centrifuged at 1500 rpm for 5 min. A total of 2 × 10^4^ cells grown (1:1) in 6 well plates containing DMEM supplemented with penicillin, FBS was incubated at 37 °C in a CO_2_ incubator. Carbocyanine dyes labeled hPMSCs, and HepG2 cells were labeled and seeded alone as a control. The fusion of both cells was monitored for 21 days to see the interaction and cell death. On day 8, the mixed cell population was determined using FACS Diva software. The parameters used in the flow cytometer are shown in Table S1.

### 2.6 Identification of the AFP and cytokine release

Specific number of the cell cultured with the different ratios (30:50, 50:50, 70:30) at the 25 cm^2^ flask and kept the cell in the incubator at 37 °C for the 48 h, 72 h. The supernatant from the co-cultured cell and also from cancer cell were collected. The assay was performed based on the protocol (Human AFP Simple Step ELISA® Kit) in the provided 96 well plates. The treated cell with the condition medium was analyzed the release of cytokine in the cell culture supernatant (Human IL-8 ELISA kit, Solarbio). The absorbance of the plate were determined through the ELISA plate reader at 450 nm.

### 2.7 Cytotoxicity by MTT assay

HepG2 cells were grown in a 96-well plate (10000 cells/ well) containing complete DMEM and incubated at 37 °C for 24 h until they reached 80–90 % confluence. HepG2 alone or co-treated with hPMSCs-CM at different ratios (35 %-50 %) were incubated for 24–72 h. Then, 5 mg/ml of MTT (Vybrant, v13154, ThermoFisher) was added in each well (10 µl), and plates were incubated for 4 h until the appearance of a purple precipitate. The medium was discarded, and added 100 μl SDS-HCL solution in each well. The plates were incubated for 4 h at CO_2_ incubator and mixed by shaker with wrapping aluminum foil. The absorbance of samples was calculated using a microplate (ELISA) reader at 570 nm wavelength.

### 2.8 Flow cytometry analysis using Annexin-V and propidium iodide

Apoptosis in HepG2 cells was detected by flow cytometry using Annexin-V and propidium iodide assay as previously described (Yuan et al., 2018). In brief, HepG2 alone or co-treated with hPMSCs or hPMSCs-CM at different ratios were incubated for 24–48 h in the CO_2_ incubator. Cells were trypsinized and washed by 1X Annexin-V binding buffer. Annexin V-APC (5 μl) was added to the cells and the mixture was kept in the dark for 16 min. Propidium iodide (1 mg/ml) solution was used as a counterstaining. Several used parameters were included in the table S1.

### 2.9 Apoptotic cell death analysis by immunofluorescence

Sterilized coverslip coated with Poly-L- lysin was kept in 6 well plates, and cells were cultured until reaching 70 % confluence. Cells were fixed by 98 % cold methanol for 10 min, permeabilized by 0.4 % triton X-100 in PBS for 10 min, and incubated with 0.1 % BSA for 2 h. The primary anti-caspase 9 antibody (1:200; ab32539, abcam) was added, and cells were incubated at 4 °C overnight. Fluorochrome conjugated secondary antibody (1:250, anti-rabbit IgG, ab150077) was added, and cells were incubated for 2 h in the dark. The mounted coverslips were examined by an immunofluorescence microscope. Hoechst 33,258 (Sigma) was used as a counterstaining dye to detect the nucleus.

### 2.10 Real-time PCR by Livak (2-ΔΔCT) method

Total RNA extraction from HepG2 cells was performed using a total RNA extraction kit (Bioer Technology, China). Concentration and purification of extracted RNA were determined by NanodropTM spectrophotometer at a wavelength A260/A280. Revert Aid first strand cDNA synthesis kit (Thermo Scientific USA, Cat no. EP0451) was used for cDNA synthesis. cDNA and master mix preparation protocol was included in table S2. The expression of apoptotic genes (Bcl-2, Bax, β-catenin) and the hPMSCs marker (Nanog) after treatment were measured by quantitative real-time PCR (qRT-PCR) using TB Green TM Premix Ex Taq (TAKARA BIO Inc.) following the manufacture instruction. Table I shows the human primers used in this study. GAPDH was used as a reference gene. Each sample was run in triplicate. Livak (2-ΔΔCT) method was used to quantify each gene expression.

### 2.11 Western blotting

Total protein from HepG2 was obtained via using RIPA cell lysis buffer. Protein concentration was determined by a Nano drop spectrophotometer, and 20 μg protein samples were separated by 10 % SDS-PAGE, transferred to the PVDF membranes, and washed with TBS for 5 min. Following the addition of blocking buffer, anti-caspase 9 (1:2500, ab32539, abcam), anti-caspase 3 (1:1000, k009567p, solar bioscience) and anti-β-actin (1:2500, k200058m, solar bioscience) primary antibodies were added, and membranes were incubated overnight at 4 °C. The membranes were then incubated with the secondary antibody (1:5000, anti-rabbit IgG, 31460) for 2 h followed by HRP colorimetric substrate for 30 min at room temperature. Digital image software (iB-right™ CL1500 Imaging System, Invitrogen) was used for band quantification.

### 2.12 Immuno-phenotyping for extrinsic and intrinsic death measurement

One million (HepG2) cells were cultured in a 25 cm^2^ flask and treated by using 90 % condition medium. The cell without treatment was also cultured in a 25 cm^2^ flask considered as control. Two million HepG2 cells were calculated and suspended in cold PBS, FCS, and kept for 5 min at room temperature. Primary antibodies were diluted anti-caspase 9, anti-caspase 3 (1:250), and mixed with 100ul of cell-containing liquid. The tubes were incubated for 30 min at 4°C in dark. Cells were washed three times for 5 min and fluorochrome-conjugated secondary antibody (ab96899, Abcam) were diluted (1:300) mixed to the cell by pipetting, incubated at dark. Cells were examined by Navies EX flow cytometer (10 colors) after 48 h of the treatment and incubation.

### 2.13 Cell cycle analysis

The effect of hPMSCs and hPMSCs-CM on HepG2 cell cycle was determined by flow cytometry as previously described (Huwaikem et al., 2021). In brief, HepG2 alone or co-treated with hPMSCs or hPMSCs-CM at different ratios were incubated for 24–48 h in the CO_2_ incubator. Cells were collected, fixed with 70 % cold ethanol, stored at −20 °C for 24 h, and washed with PBS. Propidium iodide (PI) solution (0.02 mg/ml) and 200 μg/ml RNAse were provided, and cells were incubated in the dark for 1 h. Cells were examined by FACS (BD FACSCanto™), and further evaluated by BD FACS_Diva™ Software.

### 2.14 In vitro scratch assay

HepG2 cells were plated (1 × 10^5^ cells /well) in DMEM till 75 % confluence, and a scratch was made by a yellow tip at the middle of each well. Fresh media containing hPMSCs or hPMSCs-CM at different ratios were added, and cells were re-incubated for 24–48 h. Images have been captured after 0, 24, and 48 h. HepG2 migration was estimated as previously detailed (Elsayed et al., 2020).

### 2.15 Statistical analysis

All data were expressed as mean ± standard error of mean (SEM). The difference between the groups was evaluated by one-way analysis of variance using Graph Pad prism, 7.0 software. The individual comparisons were done by Duncan's multiple range test (DMRT). Values were considered statistically significant when p < 0.05.

## 3 Results:

### 3.1 HepG2, hPMSCs morphology, fusion and inhibition

**Fig. 1:**
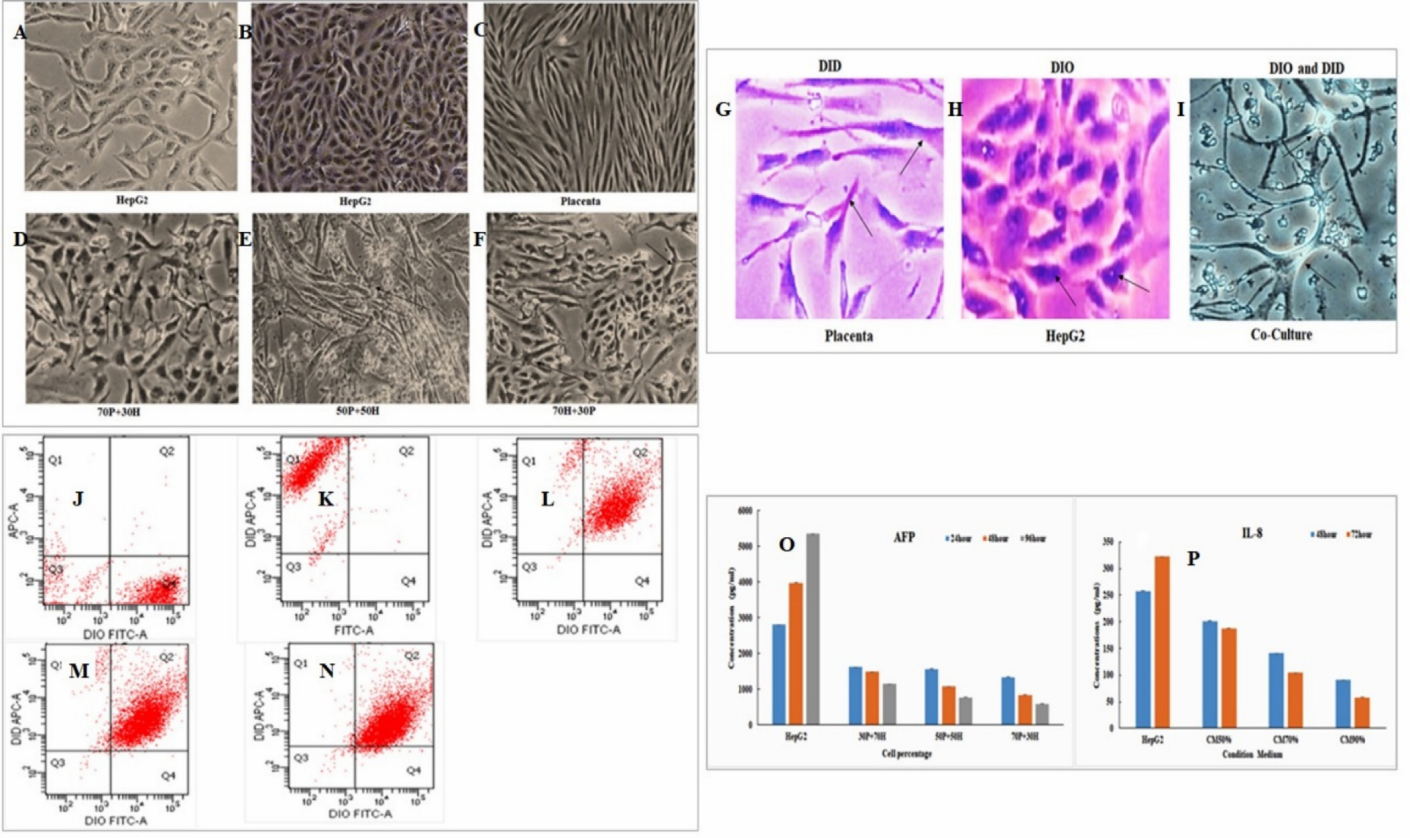
Morphology of both the untreated HepG2 after 24 h (A), 48 h (B) incubation, and hPMSCs (Placenta) after 72 h (C) incubation. Morphology of HepG2 cell line following treatment with hPMSCs at different ratios (D,E,F). Arrows indicate the point of interaction between hPMSCs and HepG2. All images were taken by immunofluorescence microscope at 4X magnification. 70P = 70 % placenta, 30H = 30 % HepG2, 50P = 50 % placenta, 50H = 50 % HepG2. Co-culture analysis of both placenta and HepG2 cell line through DIO and DID staining kit (G: placenta, H: HepG2, I: Co-culture) and using the flow cytometry (J: Placenta stain, K: HepG2, L: 70 % hPMSCs and 30 % HepG2 shows 87.5 % cell fusion efficiency, M: 50 % hPMSCs and 50 % HepG2 shows 91.25 % cell fusion efficiency, and (N) 30 % hPMSCs and 70 % HepG2 shows 89.70 % cell fusion efficiency. Cytokine and protein release through co-culture and condition medium (O) AFP production in the presence of hPMSC cell line (P) The IL-8 production was reduced in case of using condition medium.

The untreated HepG2 appeared oval, while hPMSCs displayed fibroblast-like features with extended cytoplasmic processes under the phase-contrast microscope (Fig. 1 A-C). The isolation of hPMSCs was confirmed by the Nanog gene expression, which will be detailed later. On the other hand, HepG2 treated with hPMSCs at different ratios (30P:70H, 50P:50P, and 70P:30H) demonstrated progressive morphological changes with some cells being enlarged and most of them were shrunken and encountered a higher death rate and fusion (interaction) with hPMSCs (Fig. 1D-F).

The efficiency of this fusion was confirmed by DID/DIO cell fusion assay using the phase-contrast microscope and flow cytometry. After 72 h incubation, most cancer cells died, and the merged (HepG2 + hPMSCs) cells, which contained DIO and DID, were detected (Fig. 1 G.H.I and Fig. S1). After 8 days, co-treatment with 50 % hPMSCs and 30 % HepG2 exhibited 87 ± 1.2 % cell fusion efficiency, while that of 50 % hPMSCs and 50 % HepG2 was 91 ± 0.4 % and that of 30 % hPMSCs and 70 % HepG2 was 89.5 ± 0.5 % (Fig. 1 L.M.N).

The inhibition of HepG2 cells gradually increased from D12 to D21. In contrast, the hPMSCs gradually increased and reached the maximum number on D21 (Fig. S2). The protein concentration level were determined in the supernatant of hPMSCs (negative control), HepG2 cells (positive control), and HepG2 cells co-cultured with PMSCs or supplemented using condition medium of the hPMSC. The Placenta derived supernatant has not expressed any AFP. The AFP level were decreased in comparison to the HepG2 through co-culturing of the HepG2 and hPMSC at different ratios (Fig. 1 O). On the other hand, the level of cytokine (IL-8) level thorough the treatment of the condition medium has been increased with the time period. The expression of the pro inflammatory cytokine IL-6 has been decreased in compared to the untreated group and the condition medium reduced the secretion of cytokine (Fig. 1 P).

### 3.2 Effect of hPMSCs-CM on HepG2 proliferation

**Fig. 2:**
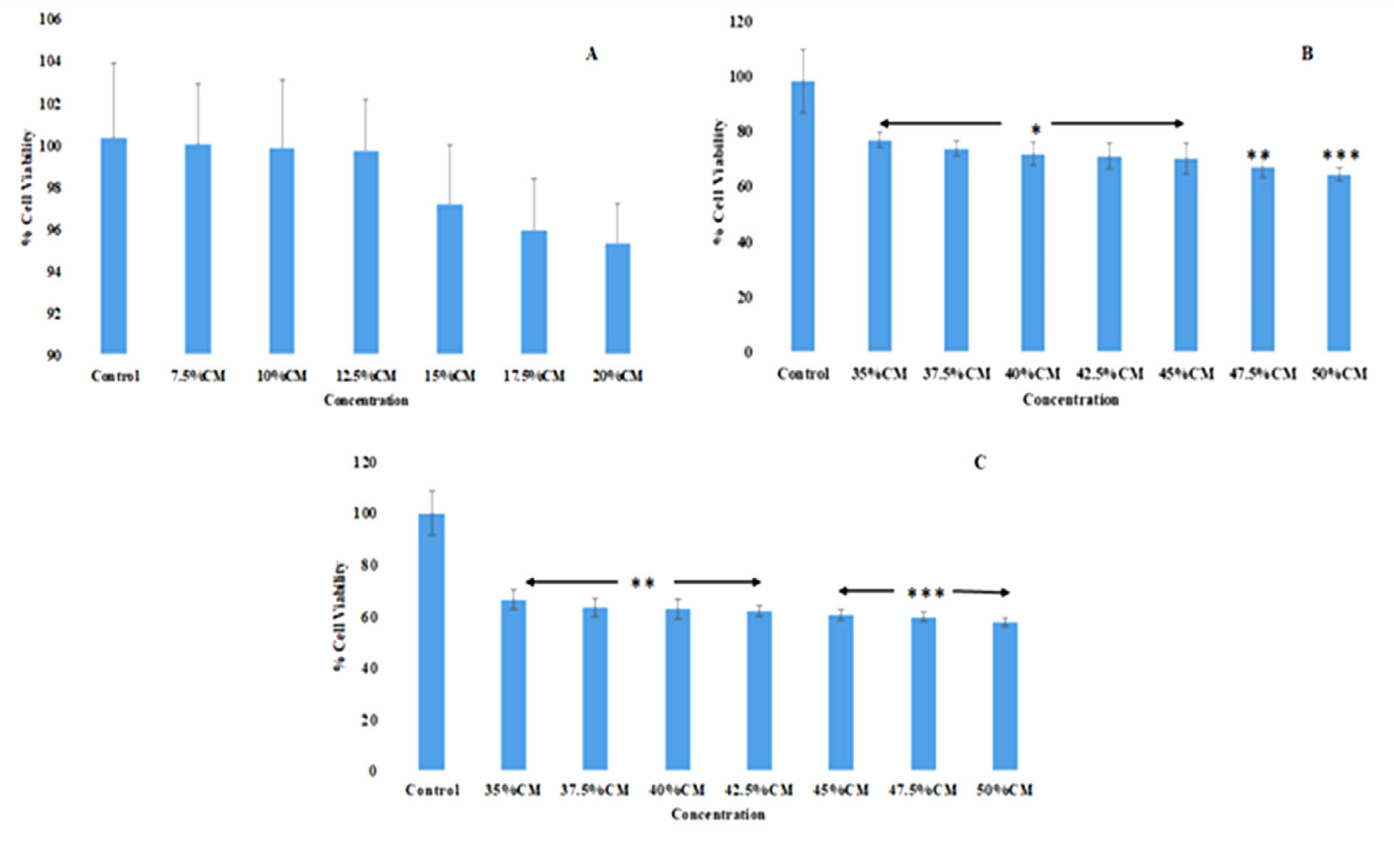
Cytotoxicity assays show the effect of hPMSCs-CM (7.5–20 %) (a) and (35–50 %) (b) on cell viability of HepG2 following incubation for 24 h, and 48 h (c). All the values were identified as mean % viability ± SEM from the absorbance of three replicates, n = 5. * P < 0.05, ** P < 0.01, and *** P < 0.001 comparing to the control (untreated) group.

MTT assay showed a dose-dependent cytotoxic effect of hPMSCs-CM at a concentration ranged from 35 % to 50 % on HepG2 compared to the control (untreated) HepG2 (Fig. 2). This cytotoxic effect increased gradually with the incubation time and the concentration of hPMSCs-CM in a way that the highest cytotoxic effect was found in HepG2 cells treated with 50 % hPMSCs-CM at 48 h.

### 3.3 HPMSCS and hPMSCs-CM induce apoptosis in HepG2

Apoptosis in HepG2 cells was detected by flow cytometry using Annexin V. Co-culture of HepG2 and hPMSCs cells induced apoptosis of cancer cells following 24–48 h incubation (Table II, Fig. 6, Fig. 7). After 24 h incubation, the treatment ratio of 70 % HepG2 cells and 30 % hPMSCs resulted in 15.3 % apoptosis which was increased to 18.5 % in 50 %H + 50 %P co-culture and reached the maximum (40.1 %) at 30 %H + 70 %P cell ratio. After 48 h, the apoptotic rate was 22.1 %, 22.5 %, and 36.1 % following treatment with 70 % H + 30 %P, 50 %H + 50 %P, and 30 % H + 70 %P, respectively. The number of cell death in co-culture might make confusion for which type of cell death were predominant, the number of placenta stem cells percentage increased the number of HepG2 death raised. As the cell fusion shown the number of placenta cell increased and qRT-PCR results also shown the increased stem cell marker ratio compared to the control.

**Fig. 3:**
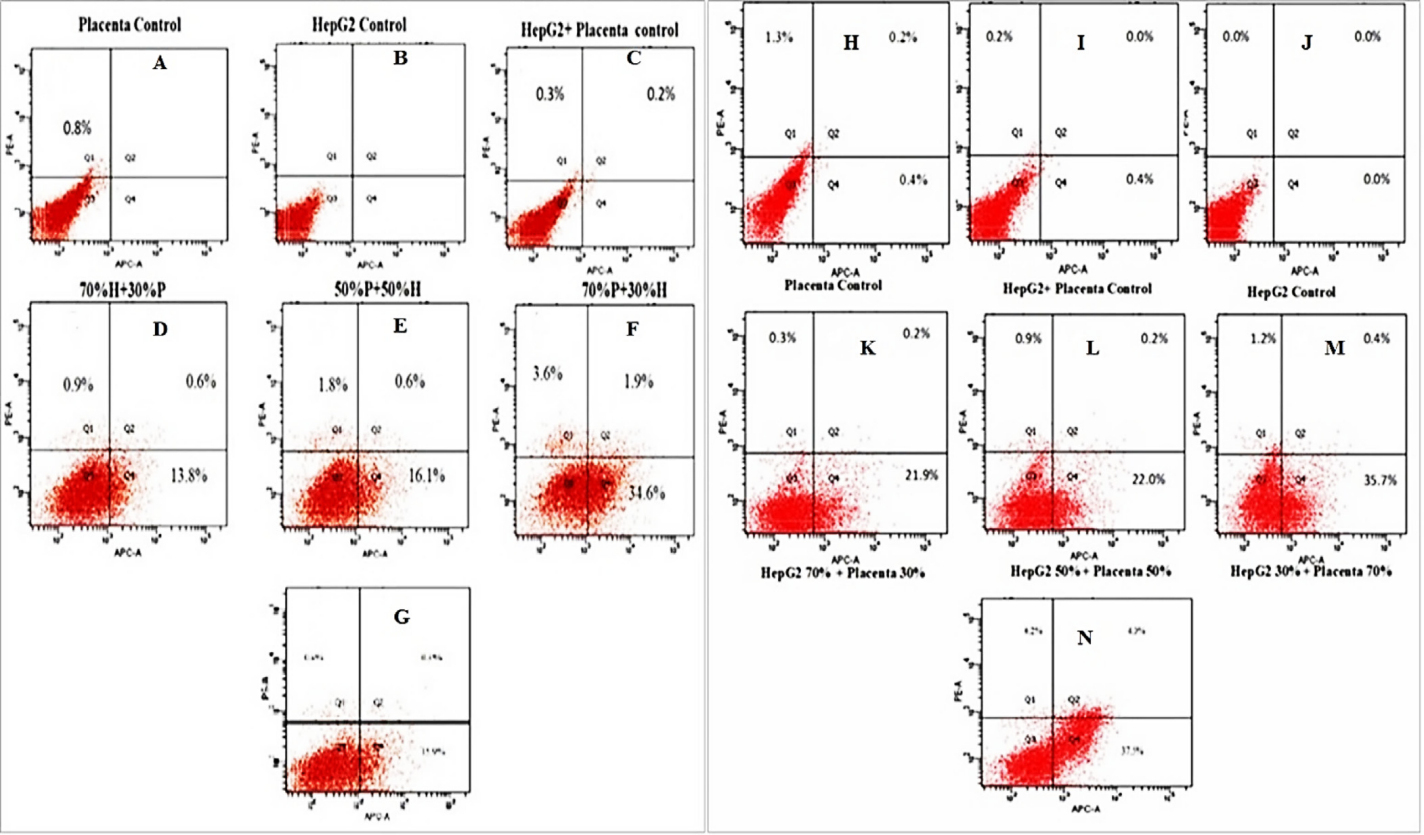
Flow cytometer scatter plot showing the presence of apoptotic cells following treatment of HepG2 with hPMSCs and hPMSCs-CM for 24 h and 48 h A) control (untreated) hP-MSCs, B) control (untreated) HepG2, C) co-culture (hPMSCs + HepG2) control before incubation, D) 70 % HepG2 and 30 % hPMSCs (Placenta), E) 50 % HepG2 and 50 % hPMSCs, F) 30 % HepG2 and 70 % hP-MSCs, and G) hPMSCs-CM. For 48 h of treatment and co-culture H) control (untreated) hP-MSCs, I) co-culture (hPMSCs + HepG2) control before incubation, J) control (untreated) HepG2, K) 70 % HepG2 and 30 % hPMSCs (Placenta), L) 50 % HepG2 and 50 % hPMSCs, M) 30 % HepG2 and 70 % hPMSCs, and N) hPMSCs-CM. The cells were stained with AnnexinV-APC/PI and analysed by FACS.

Moreover, the addition of hPMSCs-CM gradually increased the apoptotic rate in HepG2 cells (Fig. 3). Treatment with hPMSCs-CM resulted in 16.8 % and 40.5 % cell death after 24 h and 48 h, respectively.

**Fig. 4:**
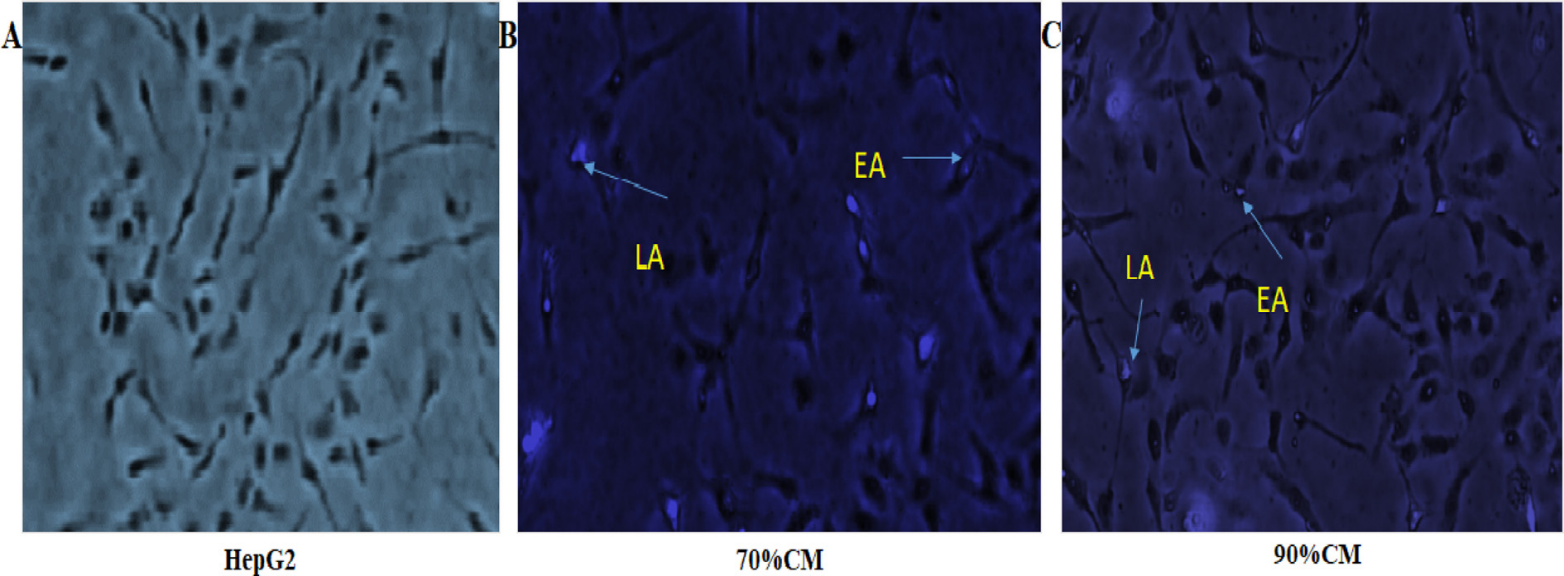
Effect of hPMSCs-CM on HepG2 apoptosis as measured by an immunofluorescence test for caspase 9. After 24 h of incubation, hPMSCs-CM was applied to the cell. (A) HepG2 cells without any treatment, (B) the cells were treated with the 70% CM, (C) the cells were treated by 90% CM. Early (EA) and late (LA) apoptotic cell death were seen by arrows. Early apoptotic cells demonstrated that apoptosis had been initiated within the cell, whereas late apoptosis took place as a result of membrane fragmentation. All images were captured using a 4x phase contrast microscope.

For further confirmation, immunofluorescent microscope was used to detect and differentiate the early and late apoptotic cells following caspase-9 immunostaining. In this assay, untreated cells had a uniform colored nucleus, with intact nuclear membrane. Following treatment of HepG2 cells with hPMSCs-CM, several HepG2 cells were underwent early and late apoptosis (Fig. 8). The early apoptotic cells showed bright green color nuclei whereas late apoptotic cell death brighter nuclei through chromatin condensation (YP et al., 2016). The late apoptotic cell death were identified by fragmentation of cells into a number of membrane-bound globules (apoptotic bodies). The death of HepG2 was dose dependent manner based on the concentration of hPMSCs-CM (Fig. 4). More apoptotic cell death photos were provided in the supplementary file (Fig. S3).

### 3.4 Effect of hPMSCs and hPMSCs-CM on apoptotic and stemness markers

**Fig. 5:**
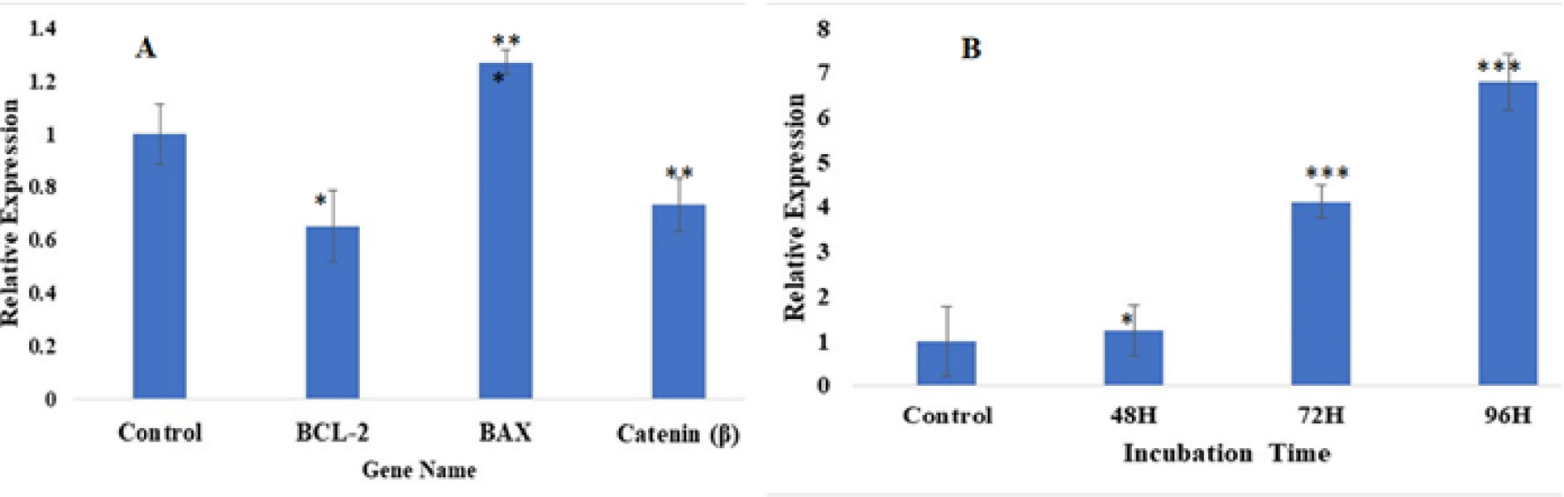
The qPCR analysis of apoptotic genes (Bcl-2, Bax and β catenin) in HepG2 cells and placenta stem cell marker (Nanog) in hPMSCs. (A) The HepG2 cells were treated by hPMSCs-CM for 24 h and identified the expression of (Bcl-2, Bax and β catenin). (B) In co-culture both hPMSCs and HepG2 incubated 48–96 h and identified the Nanog expression. GAPDH used as a control in the relative expression of other gene. * All the values were identified as mean ± SEM, n = 5. * P < 0.05, ** P < 0.01, and *** P < 0.001 comparing to the control (untreated) group.

The expression of the apoptotic gene (Bax) and anti-apoptotic genes (Bcl-2, and β-catenin) in HepG2 following treatment with hPMSCs-CM was detected using qPCR. Treatment with hPMSCs-CM significantly increased the expression of Bax but significantly decreased the expression of Bcl-2, and β-catenin in HepG2 as compared to control (untreated) cells (Fig. 5A). On the other hand, the expression of Nanog significantly and gradually increased with the increasing incubation time (Fig. 5B).

**Fig. 6:**
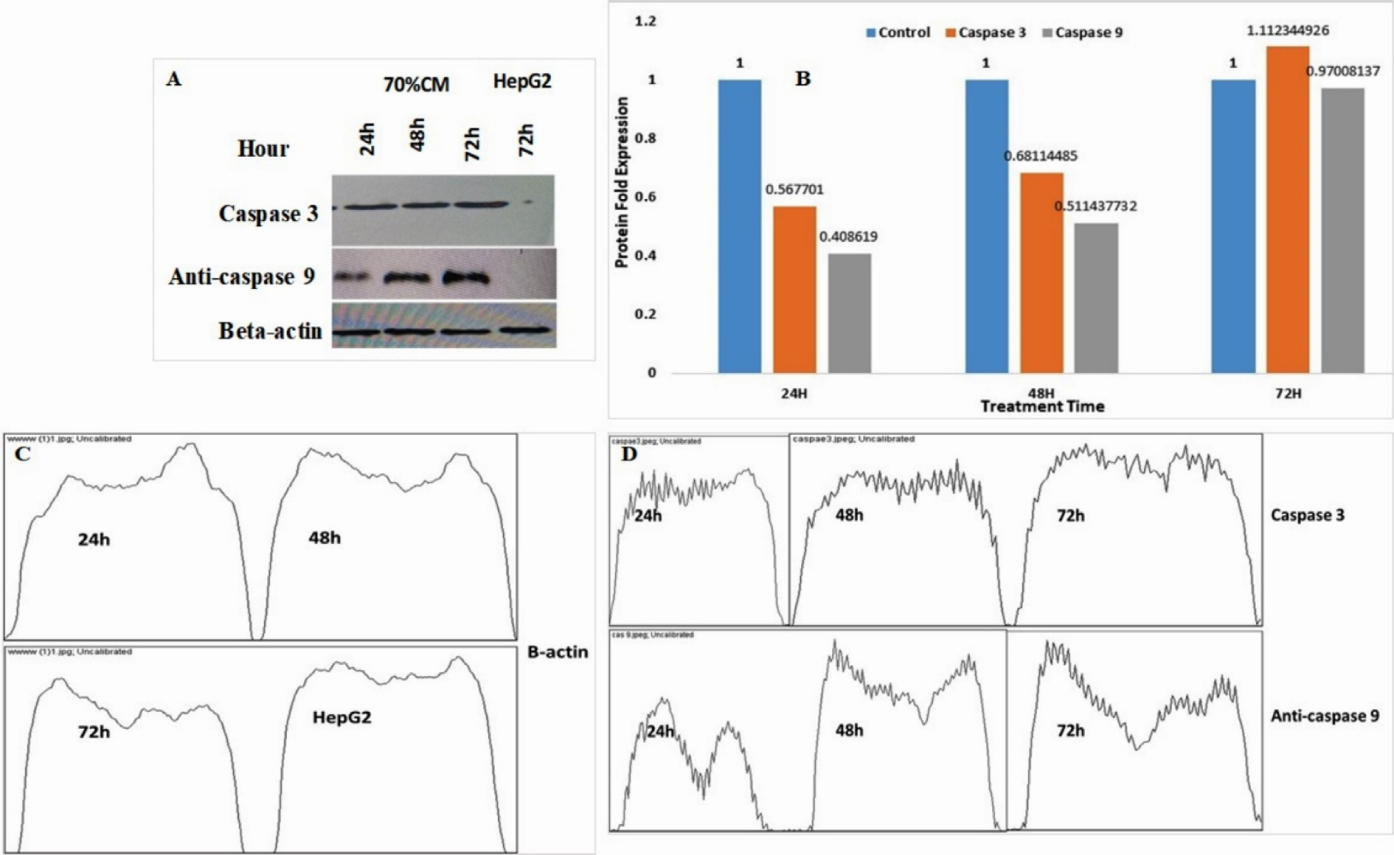
Western blot analysis of caspase-9 and caspase-3 protein expression over treated HepG2 (A). Hepatocellular carcinoma cells were treated with 70 % of hPMSCs-CM for 24 h, 48 h, 72 h. Cell lysate were collected by using the RIPA buffer and protein expression were determined by using 10 % SDS-PAGE gel (B). Control (untreated) cells showed no expression. β-actin was used as control (C). The immunoblot image were analyzed by using image J software (D).

Regarding the protein level, the effect of hPMSCs-CM on the expression of caspase 9 and caspase 3 protein was investigated by western blot (Fig. S4, Table S3). Caspase 9 and caspase 3 were expressed in HepG2 cells treated with 70 % hPMSCs-CM, however no expression was detected in control (untreated) HepG2 cells (Fig. 6).

**Fig. 7:**
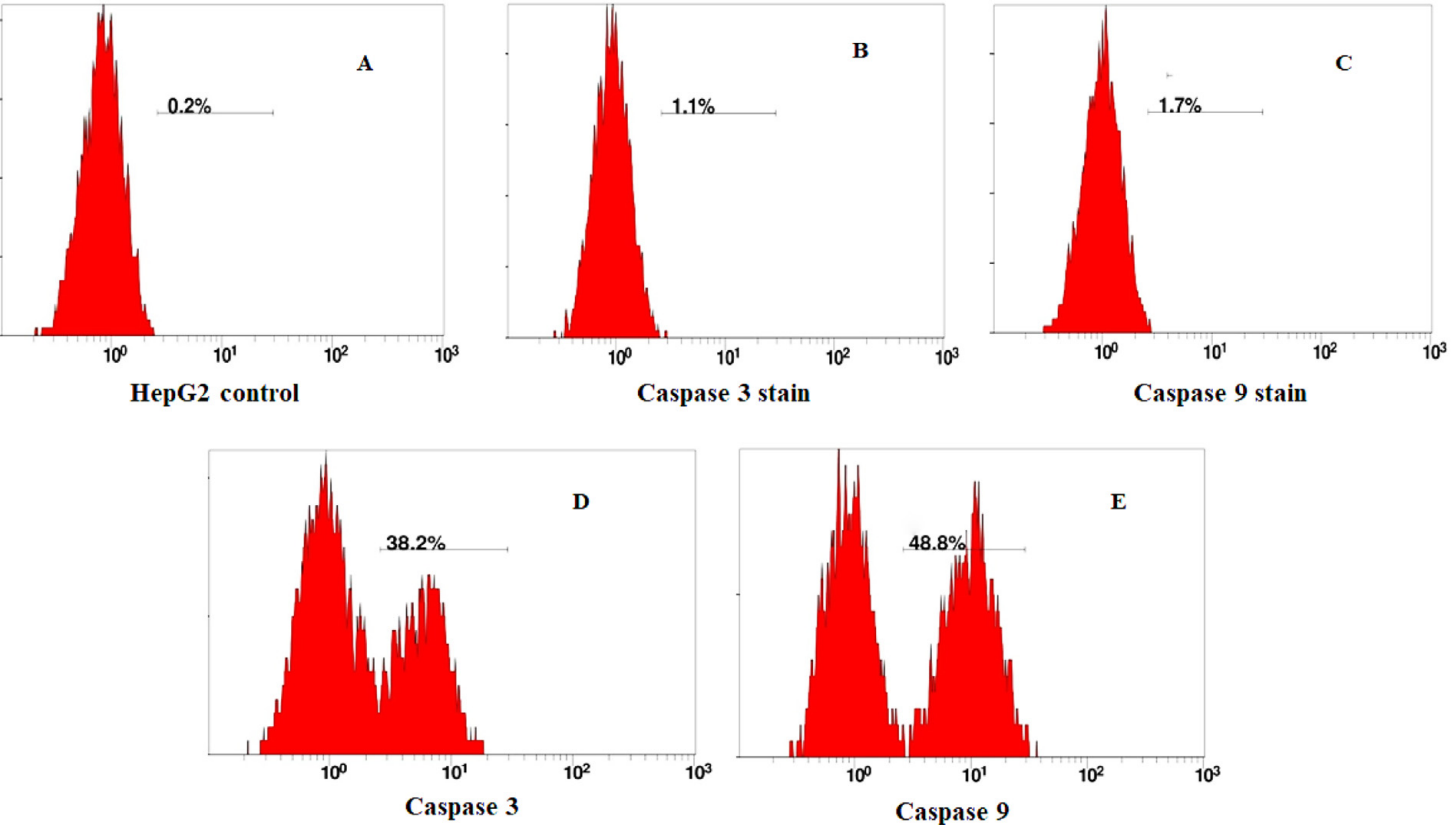
Apoptosis detection after staining with caspase-3, caspase-9 antibodies using the flow cytometry. Cells were incubated for 48 h in CO_2_ incubator after treatment with condition medium. (A) HepG2 were used a control without any antibodies (B) Caspase-3 was used to stain HepG2 cells without treatment (C) Caspase-9 staining without treatment of condition medium (D) The presence of active caspase-3 were detected at 38.2 % (E) Caspase-9 activated in the cell treatment were more (48.8 %) than caspase-3.

The expression of an extrinsic and intrinsic marker through treatment of HepG2 cells were shown in Fig. 7. Treatment of cancer cells in the presence of 90 % CM showed the activation of caspase-3 about 38.2 % and indicated the early stage of apoptosis. However, the apoptosis activation by the caspase-9 was increased by about 48.8 %. The stain HepG2 cells were indicated the accuracy of apoptosis (Fig. 7 B-C) without treatment.

### 3.5 Effect of hPMSCs and hPMSCs-CM on HepG2 cell cycle

**Fig. 8:**
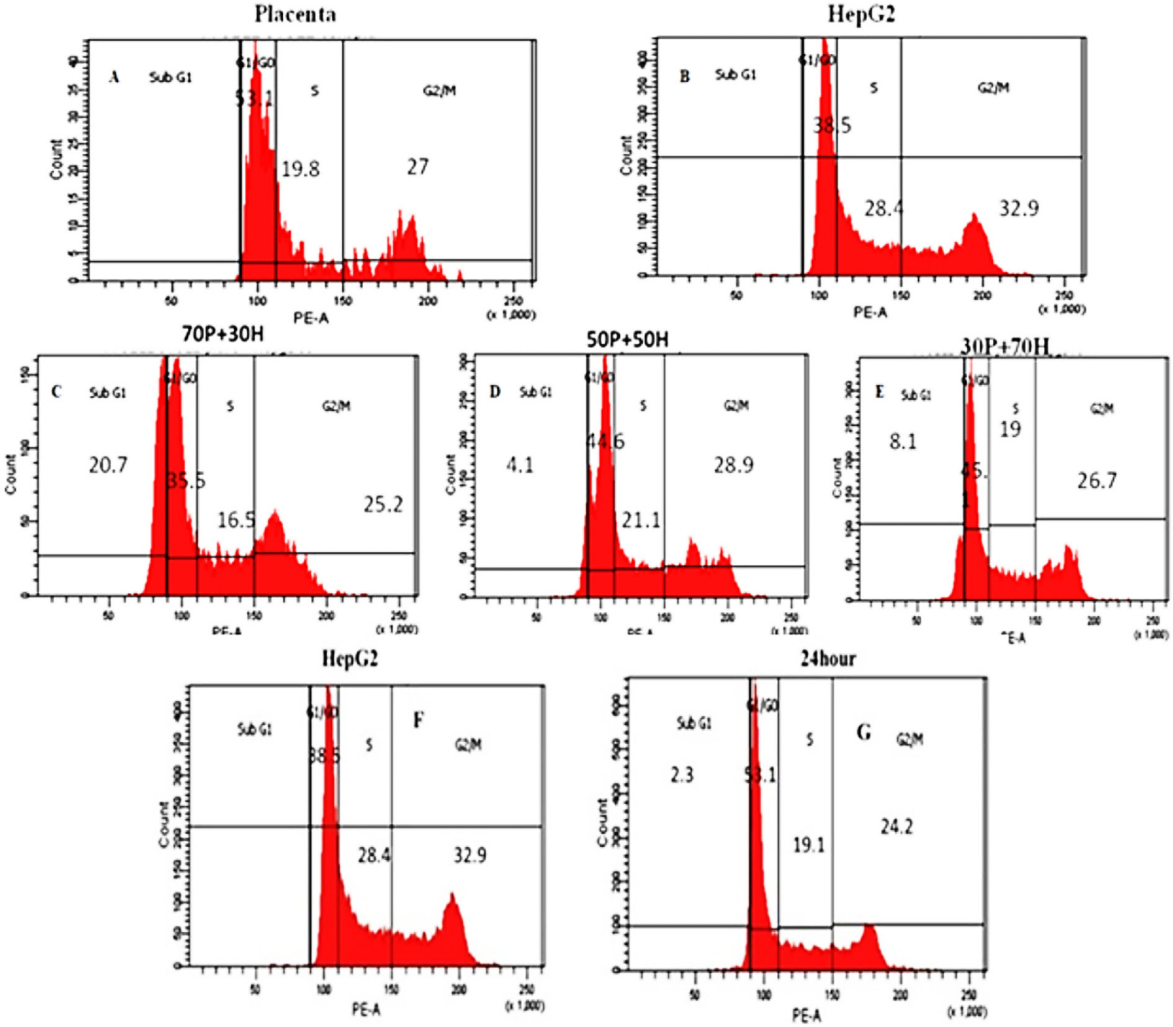
HPMSCs and hPMSCs-CM effect on HepG2 cell cycle as detected by flow cytometry. HepG2 and placenta were used as a control to see the growth cycle (A, B). the treatment of cells with several ratios of placenta cell (C, D, E). following the incubation of 24 h, the cell death was measured in 90 % condition medium (G) and untreated cells were calculated as control (F).

The sub_G1 phase increased gradually with the increasing of hPMSCs ratio (70,50,30) which means that the number of apoptotic cells increased compared to the control cells which lacked the sub_G1 phase (Fig. 8). HepG2 growth was arrested in the G0/G1 phase flowing treatment with hPMSCs and hPMSCs-CM compared to the control (untreated) cells.

### 3.6 Effect of hPMSCs-CM on migration of HepG2

HepG2 migration in the control (untreated) group took 24 h to fulfill the half of scratch area and after 48 h of incubation cells migrated into the whole scratch area. To determine the effect of hPMSCs-CM on HepG2 migration, three concentrations of CM (50 %, 70 %, 90 %) were used. The inhibition of migration was lowest for 50 %CM after 24 h (Fig. S5). At higher percentage (90 %CM), HepG2 migration was markedly inhibited as compared to lower concentrations. Comparing the two time points, the rate of migration was higher after 24 h than 48 h.

## 4 Discussion

The differential effects of hPMSCs and their conditioned media (CM) on HepG2 cells have not been elucidated yet. MSCs could directly kill HepG2 or indirectly through some factors found in their CM. Thus, this study was conducted to compare the anticancer effects of hPMSCs and hPMSCs-CM on HepG2. The main findings of this study were the presence of a higher anticancer potential against HepG2 for CM than hPMSCs. This effect was also dose dependent, where notable morphological changes, HepG2 cell growth arrest, maximum cell death, and highest inhibition of migration were observed in case of higher percentage (90 %CM).

The present study revealed that treatment of HepG2 (H) with hPMSCs (P) at different ratios (30P:70H, 50P:50P, and 70P:30H) altered HepG2 shape and induced their death. We also noticed cell fusion between both cell type as confirmed by DID/DIO cell fusion assay and flow cytometry. The rate of HepG2 cell death increased gradually with increasing the number of fused (merged) cells and incubation time. The highest number of dead HepG2 and merged cells was noticed in 30 % hPMSCs and 70 % HepG2 combination on D21. On the other hand, treatment with hPMSCs-CM resulted in a dose-dependent cytotoxic effect on HepG2 with highest cytotoxic effect for 50 % hPMSCs-CM at 72 h. Taken together, HepG2 death might be due to either the direct contact between hPMSCs and HepG2 (as revealed by cell fusion) or indirect through the release of chemokines, cytokines and other inflammatory chemicals from hPMSCs into their CM (Sasportas et al., 2009). Additionally, MSCs-CM has anti-tumor capabilities to inhibit cancer cell proliferation. Human bone marrow (hBM)-derived MSCs-CM can inhibit HepG2 cell proliferation through targeting Wnt signaling pathway (Rong et al., 2019). HBMMSC-CM can also synergistically potentiate the anti-HepG2 effect of sorafenib (Seyhoun et al., 2019b).

Our results revealed that hPMSCs and hPMSCs-CM induced HepG2 cell death through apoptosis as detected by flow cytometry using Annexin-V, by caspase 9 immunofluorescent assay, by qPCR (for detection of Bax, Bcl-2, and β-catenin genes), and by western blot (for detection of caspase-9, caspase-3 protein). The highest apoptotic effect was noticed following treatment of HepG2 cells by highest ratio/concentration of hPMSCs and hPMSCs-CM. Thus, both 30 %HepG2 + 70 % hPMSCs and 90 % hPMSCs-CM showed the highest apoptotic rate. Subsequently, this infer that the death of HepG2 was dose dependent based on the concentration of hPMSCs and its CM. In support, MSCs inhibit Bcl2, β‑catenin, and Wnt signaling, trigger apoptosis in cancer cells (Y.-H. Wang & Scadden, 2015). Moreover, hBM-MSCs secreted factor Dkk1 induces breast cancer MCF-7 cells viability loss (C. Zhang et al., 2013).

It has been already observed that co-culturing both the adipose tissue derived msenchymal stem cell (AD-MSC) and the liver cancer cell inhibited the proliferation of the cancer cell through the upregulation of the p53 gene and also induced the apoptosis of the cancer cell line (Serhal et al., 2019). The bone marrow derived msenchymal stem cells (BM-MSC) upon co-culture with the liver carcinoma cell line were shown to induce apoptosis by releasing cytokines (Chinnadurai et al., 2021). The present study explained that hPMSCs and hPMSCs-CM had the ability to induce HepG2 cell cycle arrest. HepG2 cell growth was arrested in the G0/G1 phase following treatment with hPMSCs or hPMSCs-CM. These treatments also inhibited migration of HepG2 cells with maximum effect when using highest ratio/concentration of hPMSCs (70 %) and hPMSCs-CM (90 %). Consistent with these findings, MSCs inhibit cancer cells in the G0/G1 phase of the cell cycle of some cancer cell types (Qiao, Xu, Zhao, Zhao, et al., 2008). Liver cancer treatment is expensive and needs surgery, radiotherapy, chemotherapy, liver transplantation based on the stage of cancer. From our study, we also concluded that hPMSCs may be used to treat HCC patients but this needs further in vivo studies and clinical trials. As it has already been established that stem cells having the capability to migrate wound areas and are capable to show their action to cure disease as well as to grow new cell types. We are hopeful further in vivo research and clinical trial might open a new path to treat cancer as well as other deadly diseases such as ischemic heart attack, kidney diseases, and neurodegenerative diseases.

## 5 Conclusions

Our research work suggests that co-culture of both cells and using condition medium inhibited the HepG2 cancer growth in vitro by Wnt signaling. The apoptotic cell death was determined by flow cytometry and western blot. Destroying HepG2 cells also has been identified by the co-culture and the most important things was that number of hPMSCs were increased after 21 days. The main findings of this study were the presence of a higher anticancer potential against HepG2 for CM than hPMSCs. The condition medium was also reduced the secretion of the proinflammatory cytokine based on the percentage of treatment used. This effect was also dose dependent, where notable morphological changes, HepG2 cell growth arrest, maximum cell death, and highest inhibition of migration were observed in case of higher percentage (90 %CM). Further analysis is necessary for clear mechanism against the liver cancer and also clinical trial should consider for final development as in cancer therapy.

## Ethical approval

The collection of placenta derived stem cells and the use of hepatocellular carcinoma cell was approved by the ethical committee of King Abdulaziz University (33–15/KAU).

## Author contributions

FADM Opo designed and performed experimental work, wrote the original draft, analysis result. M Moulay and S Alkarim securitized the result, analyzed the draft time to time, and provided the direction to work, provided the motivation. G Alrefaei, NH Alsubhi, MM Rahman read the manuscript, provided the valuable information, guided to work smoothly, analyzed the data.

## Funding

The present study was supported by the Deputyship for Research & Innovation, Ministry of Education, and Deanship of Scientific Research (DSR) , King Abdulaziz University (KAU), Jeddah, Saudi Arabia for funding this research work through project number “IFPRC-115-130-2020.

## Declaration of Competing Interest

The authors declare that they have no known competing financial interests or personal relationships that could have appeared to influence the work reported in this paper.
