# Supplementary material for: Effect of Co-culturing both placenta-derived mesenchymal stem cells and their condition medium in the cancer cell (HepG2) migration, damage through apoptosis and cell cycle arrest
Source: Saudi J Biol Sci. 2022 Dec 1;30(2):103519. doi: 10.1016/j.sjbs.2022.103519 (PMC9763848; doi:10.1016/j.sjbs.2022.103519)
Supplement: Supplementary data 1 [file mmc1.docx]

**Electronic Supplemental Materials (ESM)**

**Table S1**: Parameters used in fusion of both cells during the flow cytometry analysis.

| **Number** | **Fluorescent Parameters used** | **Voltage (Volt)** |
| --- | --- | --- |
| 1 | FSC | 137 |
| 2 | SSC | 304 |
| 3 | FITC | 412 |
| 4 | PE | 521 |
| 5 | PerCP-Cy5-5 | 703 |
| 6 | PE-Cy7 | 649 |
| 7 | APC | 403 |
| 8 | APC-Cy7 | 641 |
| 9 | Pacific Blue | 530 |
| 10 | AmCyan | 612 |

**Table S2**: Master Mix for cDNA synthesis and qRT-PCR reaction protocol for analysis of gene expression.

| **cDNA Synthesis** | | **RT- PCR reaction components** | |
| --- | --- | --- | --- |
| **Components** | **Volume(µl)/each reaction** | **Components** | **Volume(**µl**)/each reaction** |
| Total RNA | 2 | TB Green Premix | 12.5 |
| Primer | 1 | PCR Forward Primer | 1 |
| Water, Nuclease free | 9 | PCR Reverse Primer | 1 |
| 5X reaction Buffer | 4 | Sterile Purified Water | 8.5 |
| RNase inhibitor | 1 | Template | 2 |
| dNTP mix | 2 | - | - |
| Revert aid M-MuLV | 1 | - | - |
| Total Volume (µl) | 20 | Total Volume (µl) | 25 |

**Table S3:** Densitometry Readings/intensity Ratio were calculated based on the immunoblot result

|  | Antibody | 24Hour | 48Hour | 72Hour |
| --- | --- | --- | --- | --- |
| Control | Actin | 72807.43 | 81835.454 | 65415.36 |
| Treated | Caspase 9 | 29750.51 | 41853.739 | 63458.22 |
| Treated | caspase 3 | 41332.89 | 55741.798 | 72764.45 |


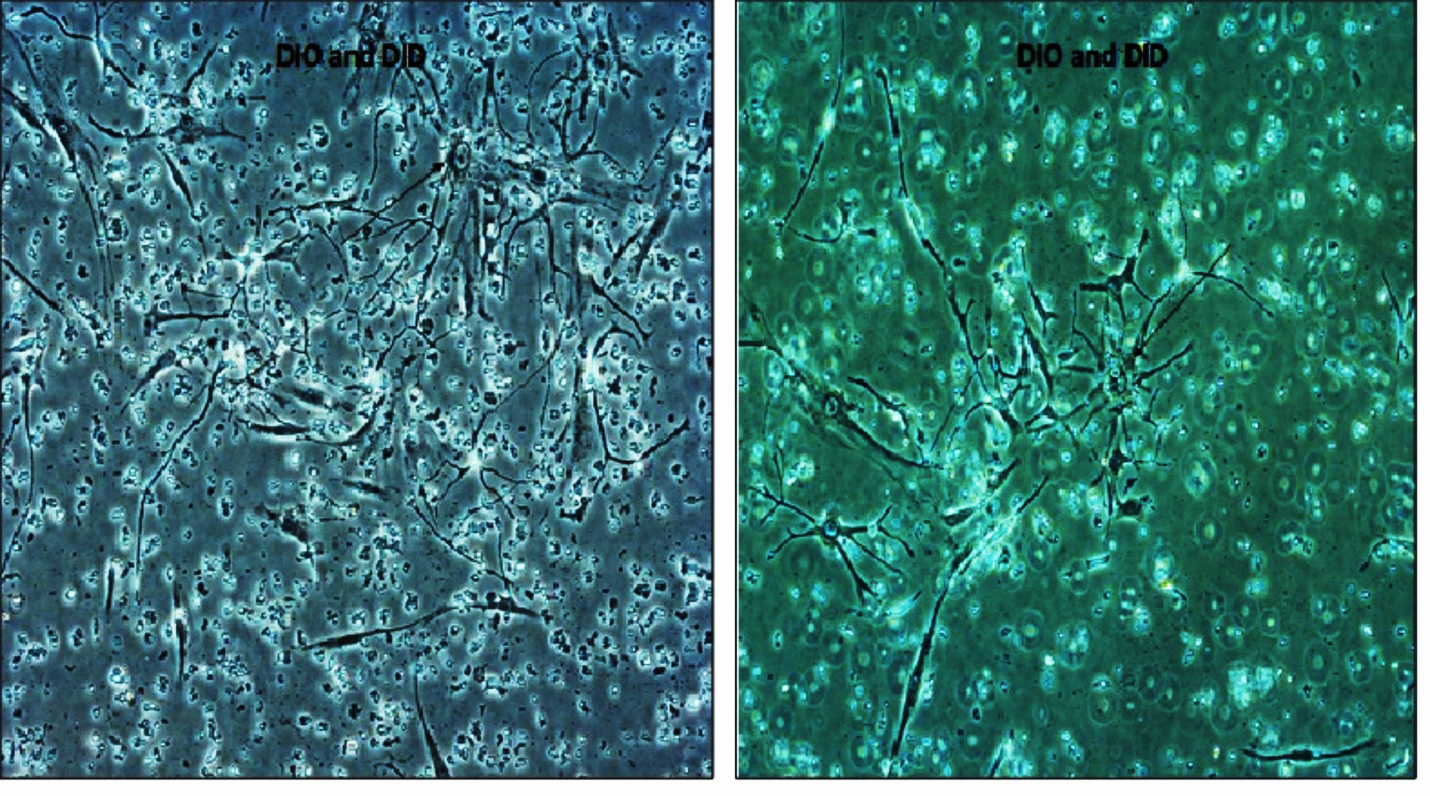


**Figure S1:** DIO and DID both color used to detect interaction between two live cells. Photos were taken by using inverted phase contrast microscope. Live cells were attached to the six well plates and dead cells were floated on the media.

**Fig. S2**. Microscopic analysis of hPMSCs and HepG2 cell fusion at days 12, 15, 18, and 21. Photos were taken by immunofluorescence microscope at 4X magnification (scale bar 100µm).


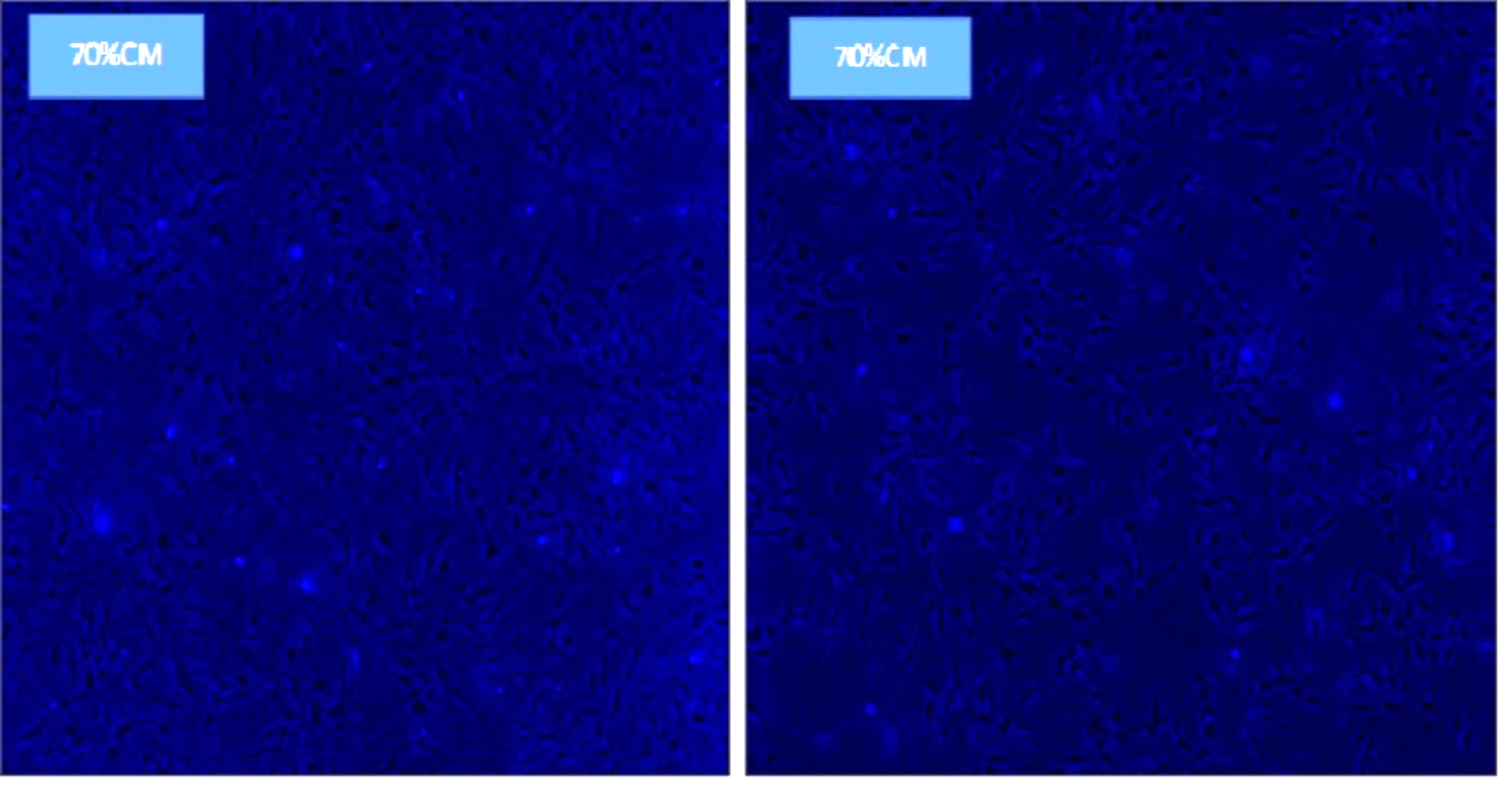

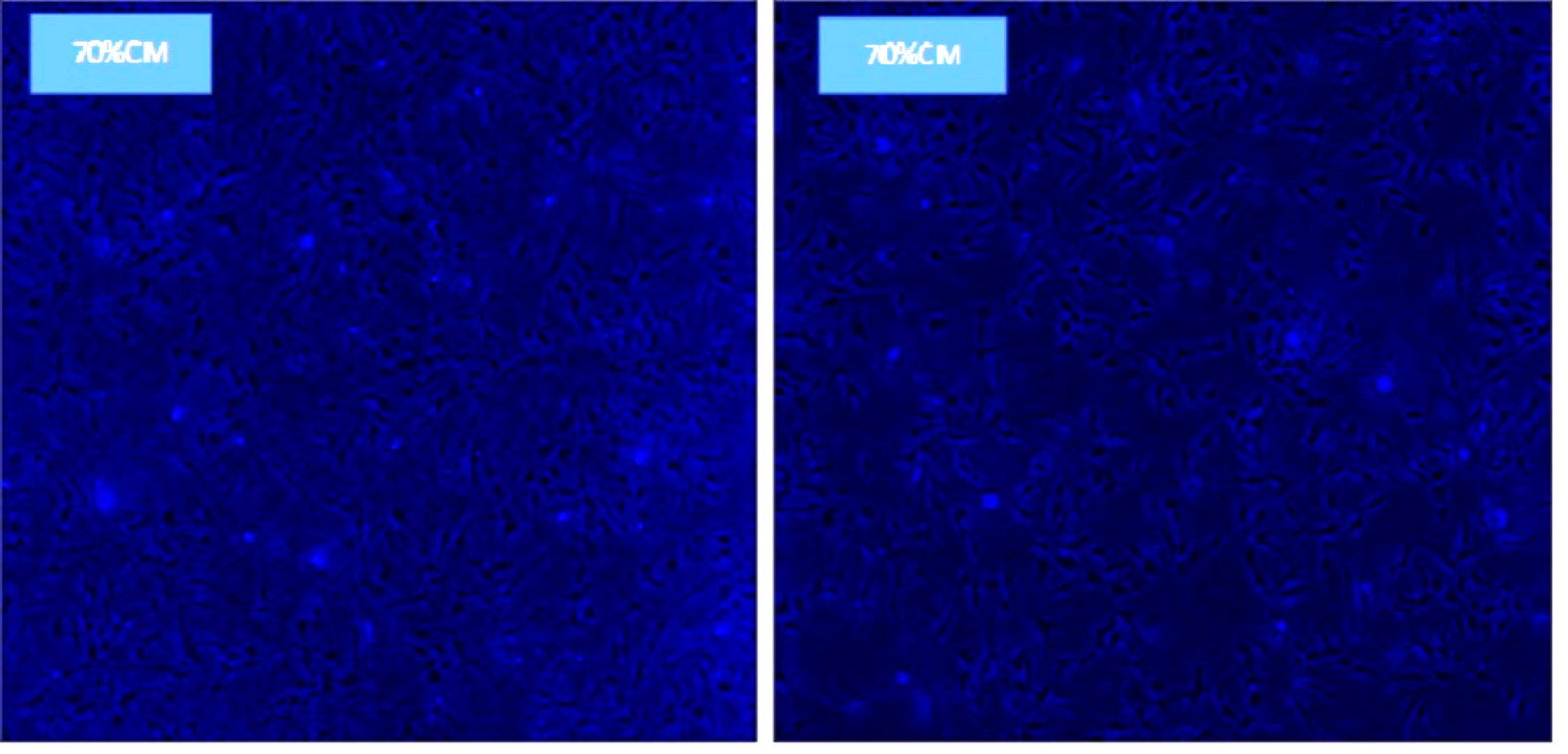


**Figure S3:** The apoptotic death of HepG2 cells based on the treatment of condition medium. Immunofluorescence study was conducted to see the apoptotic cell death after specific time period of incubation. Phase contrast microscope were used to take the photos. The death cell was emitted light were detected by fluorescence microscope

**
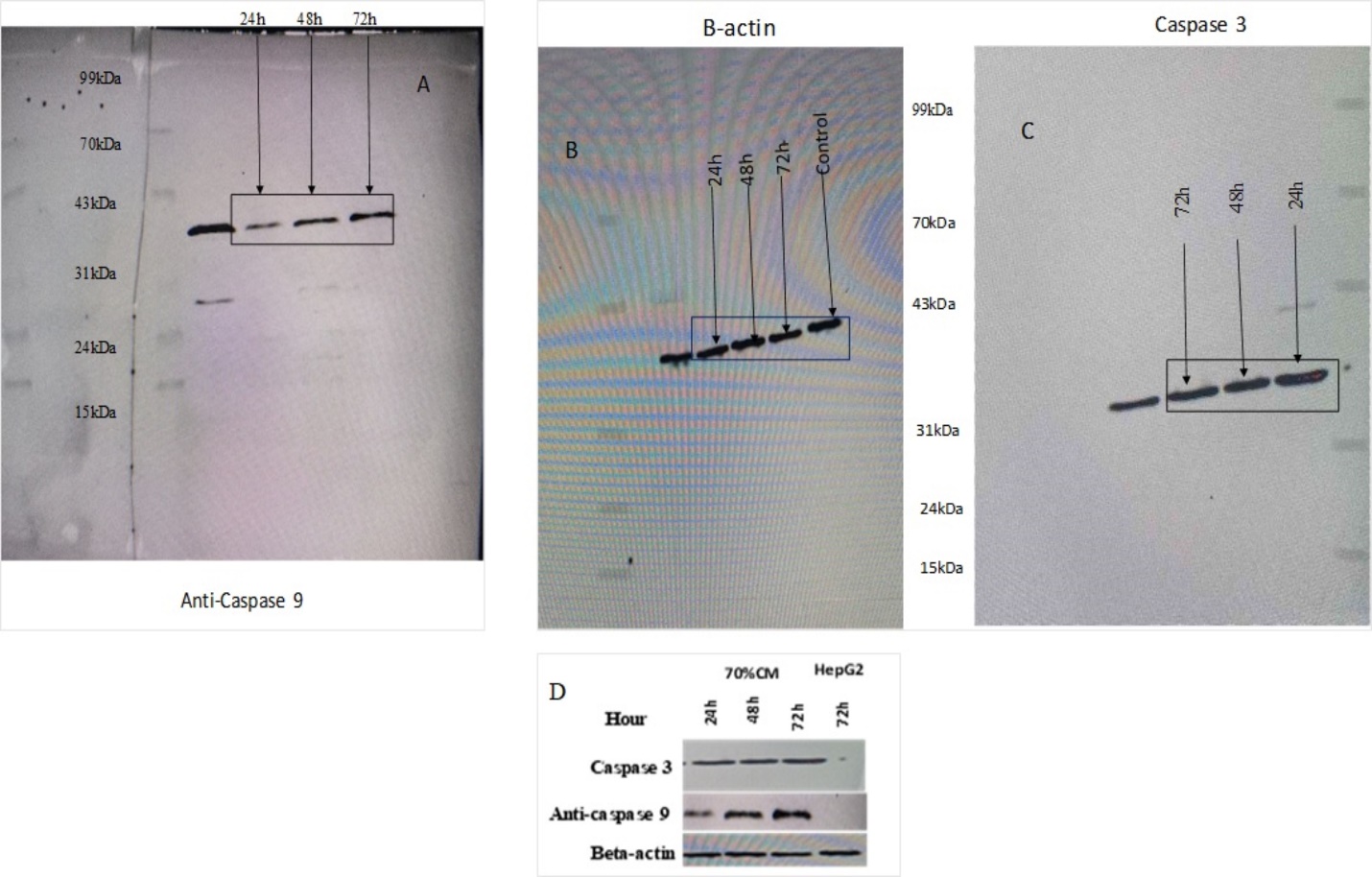
**

**Figure S4:** Non stain western blot. The photos were taken by using I bright imager (Thermo scientific). The treated HepG2 cell line with the presence of 70%CM (A, C) showed the activation of caspase pathway. (B) Same membrane was used by using the western blot stripping solution to see the control (b-actin) expression in our treated and non-treated sample. We used pre stained protein (PR1600, Solarbio, China). All the bands together showed (D) with different time periods.


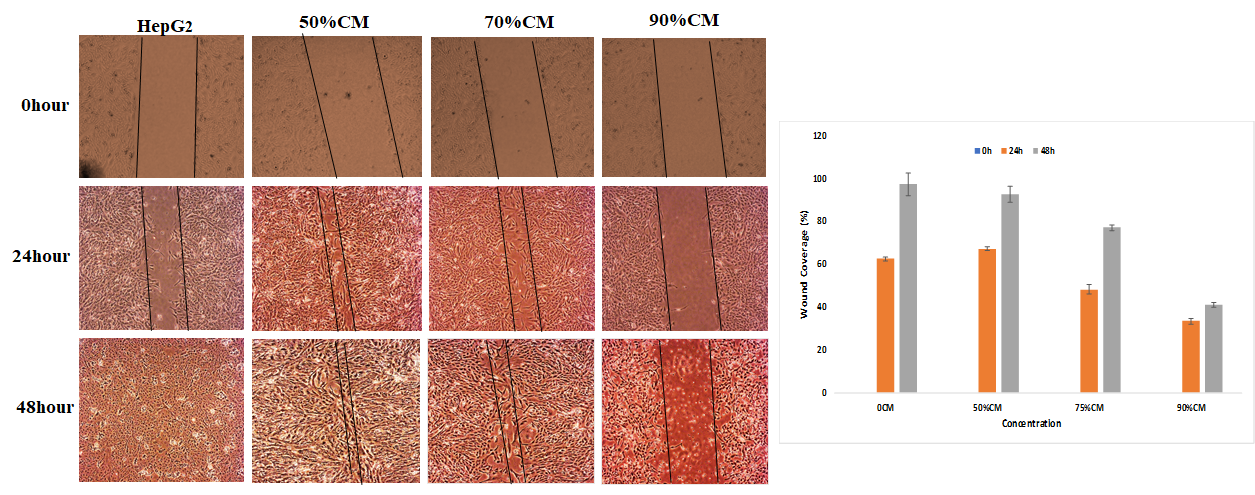


**Figure S5**: Effect of condition medium on the migration of HepG2 cells. Untreated were considered as a control, both treated and untreated kept for 48h and analyzed the rate of migration. Wound coverage mean areas were calculated using Image J software.
